# Congenital Human Cytomegalovirus and the Complement System

**DOI:** 10.3390/v17101324

**Published:** 2025-09-29

**Authors:** Andrea Canto Garon, Yujun Liu, Fenyong Liu

**Affiliations:** 1 Program in Comparative Biochemistry, University of California, Berkeley, CA 94720, USA; 2 School of Public Health, University of California, Berkeley, CA 94720, USA

**Keywords:** birth defects, complement, congenital infection, cytomegalovirus, herpesvirus, human cytomegalovirus, placenta, innate immunity

## Abstract

Congenital human cytomegalovirus (HCMV) infection is the most common vertically transmitted viral infection, and it affects 1 in 200 live births worldwide. While neonates are often asymptomatic at birth, congenital HCMV infection can result in long-term complications, including microcephaly, sensorineural hearing loss, and neurodevelopmental abnormalities. Developing antiviral strategies for the treatment and prevention of congenital HCMV infections is a global public health priority. However, licensed anti-HCMV vaccines are not yet available, and therapeutic options for use during pregnancy remain limited. The complement system is a crucial component of the innate immune system that plays essential roles in both fetal development and maternal defense against infectious pathogens. In cases of congenital HCMV infection, complement may contribute to the successful containment of the virus, but dysregulation and overactivation could concurrently drive tissue-damaging inflammation. This review discusses the known roles of the complement system in fetal development and in HCMV pathogenesis and synthesizes existing research to develop the hypothesis that a dysregulated complement system is a key mechanism in the development of congenital HCMV-related pathogenesis and neurodevelopmental sequelae. We explore how HCMV may perturb the complement system during pregnancy and use one inhibitor example to illustrate the broader potential of targeting complement in limiting disease.

## 1. Introduction

Human cytomegalovirus (HCMV), or human herpesvirus 5 (HHV-5), is a widespread β-herpesvirus [1,2]. Congenital HCMV infection is the most common vertically transmitted viral infection, affecting approximately 1 in every 200 live births globally [3,4]. HCMV congenital infection, a major cause of neonatal morbidity, can cause several complications, including pneumonia, gastrointestinal damage, and central nervous system damage, and can even lead to neonatal death [5,6]. It has been suggested that the most severe threat to fetal health arises during a primary maternal infection [1]. Most neonates are asymptomatic at birth; however, a subset experience health challenges [7,8].

These adverse birth outcomes include symptoms such as sensorineural hearing loss (the most common long-term complication), microcephaly, developmental delays, fetal growth restriction (FGR), hepatosplenomegaly, jaundice, and, in some cases, retinitis [3,7]. Congenital HCMV represents a major public health burden, standing as a leading cause of non-genetic hearing loss and neurodevelopmental disability worldwide [3,4]. Despite the prevalence and impact of the virus, there is currently no licensed vaccine available, and options for treatment are limited, especially during pregnancy [1,9,10,11].

The complement system is a fundamental component of the innate immune system, acting as a rapid defense mechanism [12,13,14]. The complement cascade is crucial for early action to control viral spread, particularly in early life when the adaptive immune system is still developing. The cascade facilitates the targeting of pathogens through opsonization and forms the membrane attack complex (MAC) [13,15]. Potent anaphylatoxins, such as C3a and C5a, further enhance this response by attracting immune cells to specific locations [12,14].

The complement system, while known for its well-established role in immune function, also plays critical roles in developmental biology [12,16]. Neural development, specifically the refinement of neural circuits and the elimination of unnecessary synapses, involves complement proteins such as C1q and C3 [17,18,19]. Complement also contributes to the development of the placenta, angiogenesis, and immune tolerance at the maternal-fetal interface. Tight regulation of complement activation is crucial during pregnancy, as misdirected or excessive activation can contribute to pregnancy complications, fetal injury, or inflammatory damage [16,17].

While the mechanisms of HCMV-induced pathology are multifactorial, the interaction of the complement system and congenital HCMV infection is a topic that is clinically relevant but remains incompletely understood. Direct evidence linking HCMV’s modulation of complement to congenital disease is scarce. Therefore, this review synthesizes existing literature from immunology, virology, and developmental biology to propose a novel, testable framework: that HCMV-mediated dysregulation of complement is a key driver of placental and fetal pathology.

The focus of this review is to explore how activation of the complement cascade contributes to immune responses against HCMV infection and how dysregulation of complement can contribute to fetal pathology, specifically impacting the developing placenta and brain. We hypothesize that HCMV infection during pregnancy leads to dysregulation of the complement system, potentially mirroring other immune-mediated diseases where the host response itself becomes the primary driver of pathology. It is hypothesized that this could occur at the maternal-fetal interface, which contributes to placental injury and adverse congenital outcomes.

## 2. Human Cytomegalovirus

HCMV is a ubiquitous human herpesvirus [1,2]. The virus can spread through several routes, including blood transfusions, organ transplantation, and close contact with infected individuals [3,20]. Most healthy individuals, when infected, are asymptomatic or experience mild symptoms, but some populations are at significantly higher risk for severe diseases [7,8]. These include immunosuppressed patients, pregnant individuals, and developing fetuses in utero [3,7,14]. Congenital HCMV infection, a major cause of neonatal morbidity, can cause several complications, including pneumonia, gastrointestinal and liver damage, and central nervous system damage, and can even lead to neonatal death [5,6]. HCMV can be transmitted from mother to child via congenital transmission before birth, exposure during delivery, or postpartum through breast milk [6,21]. Infection in these vulnerable populations can lead to severe complications, including systemic illness, multi-organ damage, and long-term neurodevelopmental deficits [3,7,8,22].

In addition to its broad tissue tropism and diverse transmission routes, HCMV is also notable for its ability to establish lifelong latency within the host [1]. Upon primary infection, the virus can persist in a dormant state within myeloid lineage cells, particularly monocytes [23,24]. This gives the virus the potential to later reactivate in immunocompromised individuals and cause recurrent disease [1]. The ability to evade surveillance by the immune system is supported further by a large set of viral genes that are dedicated to disrupting both the innate and adaptive immune responses, including complement-mediated immune responses [14,25].

Several FDA-approved compounds are currently available for the treatment of HCMV infections and associated diseases. Due to their side effects and toxicity, these compounds are not suitable for use in pregnant women, developing fetuses, or neonates [1]. These FDA-approved compounds against HCMV infection include Ganciclovir or its oral prodrug Valganciclovir, Foscarnet, and Cidofovir. These drugs function to block the enzymatic activity of HCMV DNA polymerase and inhibit viral DNA synthesis and genome replication [26,27,28]. However, the substantial toxicity of these compounds hinders their clinical usage in humans [1]. For example, neutropenia, a condition characterized by a decreased number of neutrophils, has been found to be associated with the use of Ganciclovir and Valganciclovir, with increased incidence of secondary infections [29]. Nephrotoxicity, which can cause long-term kidney injury, has been found to be associated with the use of Foscarnet and Cidofovir [30]. Moreover, these FDA-approved compounds have little effect on HCMV latent infection and cannot fully eliminate the latent viral DNA genome. These shortcomings of current anti-HCMV treatments underscore the need for safer and more effective therapeutic strategies against this virus, especially in pregnant women, developing fetuses, and neonates.

Due to the profound impacts of congenital HCMV infection on our society, the development of a vaccine for preventing and controlling the infection of HCMV is a major global public health priority [10,11]. Extensive research has been conducted on numerous anti-HCMV vaccine candidates with different designs. Promising progress has been made in understanding the anti-HCMV immune responses induced by these vaccine candidates. For example, extensive studies have focused on understanding the immune responses in humans and animals induced by various vaccine candidates with different designs, such as inactivated whole viral antigen vaccines, virus-like particle (VLP) or subviral dense bodies vaccines, recombinant protein vaccines, DNA vaccines, and vaccines based on live attenuated virus vectors [10,11,31]. Although older antiviral drugs remain the standard of care, newer and less toxic therapies (e.g., Letermovir and Maribavir) are approved for other clinical settings and are currently being investigated for the treatment of congenital HCMV. However, an FDA-approved vaccine for gestational HCMV remains elusive and is currently not available for prevention against infection and associated diseases.

## 3. The Human Complement System

### 3.1. Complement System Overview

**Figure 1:**
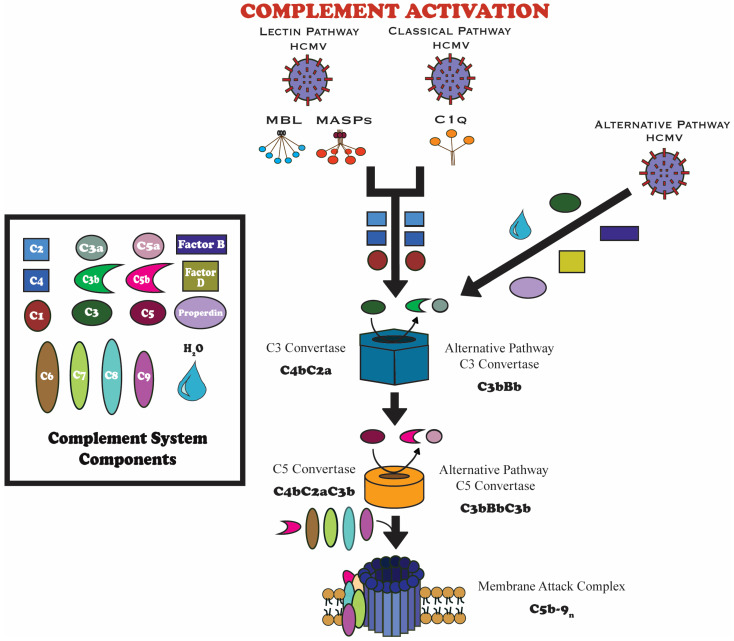
Schematic of the three complement activation pathways (classical, lectin, and alternative) [14,16]. All converge at C3 convertase, producing C5 convertase, C5a, and the MAC. The alternative pathway is shown separately due to its distinct convertase components. HCMV, like other pathogens, can initiate complement through multiple routes, including C1q, mannose-binding lectin, or spontaneous C3 hydrolysis [14,16].

The complement system is a foundational component of human innate immunity, composed of more than 30 proteins, produced primarily by the liver, that circulate in the blood or are expressed on cell surfaces [12,13,14]. The primary role of the cascade includes enhancing phagocytosis, identifying and neutralizing pathogens, and modulating inflammation. Activation of the complement system occurs via three distinct pathways: the classical, lectin, and alternative pathways [12,13]. As shown in Figure 1, these pathways ultimately converge at a central step of the cleavage of complement component C3 [15]. This convergence initiates a cascade of events that supports early immune defense, including the formation of the MAC, which disrupts pathogen membranes and induces lysis of infected cells [12,13,14].

### 3.2. Complement Pathways

The classical pathway is typically triggered when the protein C1q binds to antibodies attached to pathogens [12,13]. This interaction initiates a proteolytic sequence that cleaves complement components C4 and C2. This results in the formation of the enzyme complex C4bC2a, known as the C3 convertase (Figure 1). The C3 convertase splits C3 into two fragments: C3a, which acts as an inflammatory mediator, and C3b, which binds to the surface of the pathogen to facilitate opsonization and further progression of the cascade [12,13,14].

The lectin pathway functions similarly to the classical pathway in its downstream effects, but is initiated differently [12,13]. Activation occurs when host lectins, like mannose-binding lectin (MBL), ficolins, or collectins, bind to specific carbohydrate structures located on microbial surfaces. This interaction activates associated serine proteases, which cleave C4 and C2 to form the same C3 convertase as in the classical pathway (C4bC2a) (Figure 1) [14,15].

The alternative pathway provides consistent low-level surveillance by spontaneously activating in the blood plasma through the hydrolysis of C3 [12,13]. When C3b binds to microbial surfaces, this protein associates with factor B and is cleaved by factor D to form the alternative C3 convertase (C3bBb). This complex is stabilized by properdin and rapidly amplifies the response by generating more C3b and eventually forming the C5 convertase (C3bBbC3b), which initiates activation of the terminal pathway of the cascade (Figure 1) [14,15].

Ultimately, all three pathways converge at the activation of C5. This leads to the stepwise assembly of the MAC, a pore-forming, transmembrane complex composed of C5b, C6, C7, C8, and several copies of C9 [12,13]. This complex is embedded into target cell membranes and causes cell lysis, contributing to the clearance of foreign or infected cells (Figure 1). Although direct MAC-mediated inactivation has been demonstrated for other enveloped viruses, there is no evidence of this occurring in cases of HCMV infection. Furthermore, it is unclear whether MAC-induced lysis in HCMV infection is specific to the infected host cell or if it can also target the free virion.

## 4. Role of Complement in Fetal Development and Pregnancy

### 4.1. Complement-Mediated Neural Development

During the development of the fetal brain, the complement system plays a crucial role in synaptic pruning [17]. This process utilizes complement proteins, such as C1q and C3, to tag immature or excess synapses for elimination. Pruning is important in sculpting neural circuits into accurate and efficient systems, especially in the regions responsible for cognitive and sensory functions [32]. Additionally, complement system activity helps to further refine neural circuits by helping to guide the maturation of brain networks essential in perception, learning, consciousness, and memory. We speculate that perturbation of this highly controlled system, whether through genetic abnormality or through damage sustained from external factors such as pathogenic infection, could lead to abnormal patterns of connectivity [32]. Intriguingly, the same complement system pathways that are active during development have also been implicated in neurodegenerative diseases such as Alzheimer’s disease [33]. We hypothesize that this could suggest that inappropriate activation of complement during pregnancy could expose the fetus to molecular vulnerabilities that can result in long-term neurological consequences.

### 4.2. The Complement System in Placental Biology

Signaling from the complement cascade plays a critical role in the development of the placenta, especially during invasion of the trophoblast [34]. Complement proteins produced locally at the maternal-fetal interface are activated early in gestation, guiding immune cell recruitment and supporting vascular remodeling essential for fetal growth and development. Improper regulation of this mechanism, especially involving the potent anaphylatoxin C5a, can impair angiogenesis and contribute to impaired placental formation [34]. Proper regulation of the complement cascade is, therefore, essential for assembling a robust vascular network that ensures adequate maternal-fetal blood flow and nutrient delivery.

Additionally, dysregulation or overactivation of the cascade can lead to excessive inflammation and subsequent damage to the tissues of the placenta [35]. These perturbations are implicated in several complications during pregnancy, including preeclampsia and FGR [36]. Both of these conditions are strongly associated with placental function impairment, with the potential to cause long-term impacts on both fetal and maternal outcomes [36].

### 4.3. Balancing Immunity and Tolerance: The Role of Complement in Gestation

Immunologically speaking, pregnancy poses a unique challenge. The fetus is semi-allogeneic, meaning it carries antigens from both mother and father. In theory, this could trigger a response from the maternal immune system. The maternal immune system must tolerate the presence of a semi-allogeneic fetus without compromising its own ability to defend against infection [37]. The maternal-fetal interface relies on a highly regulated immunological environment to prevent rejection by the immune system. As shown in Table 1, fetal-derived tissues, like the placenta, express high levels of complement regulatory proteins, including CD46, CD55, and CD59 [16]. These cascade proteins help protect fetal cells from complement-mediated lysis and inflammation. They also help to sustain immune tolerance through limiting excessive activation from the complement cascade while still maintaining surveillance against infectious agents [34]. Despite its mechanistic precision, this immunological balance is inherently fragile. The complement system maintains a delicate balance within the host, acting as both a key defender against pathogens and a crucial regulator of normal developmental processes—a balance that, we propose, is dangerously tipped during congenital HCMV infection.

This model, informed by parallels with conditions like placental malaria [38], theorizes a two-pronged scenario. First, HCMV encodes complement inhibitors for self-preservation against direct complement attack. Simultaneously, the viral presence triggers an overwhelming host inflammatory response, causing collateral damage that becomes the main driver of pathology. This framework suggests that effective therapies may require a dual approach that not only targets the virus but also mitigates the host’s dysregulated immune response.

## 5. Complement Activation During Congenital HCMV Infection and Viral Modulation of Complement in Pregnancy

### 5.1. Evidence of Activation and Pathophysiological Consequences for the Fetus

Clinical studies observing inflammation-associated pregnancy complications have reported that complement components, such as C3a, were at elevated levels [39]. This suggests localized activation of the complement cascade. However, the lack of corresponding elevation in C5a points to restricted or regulated terminal pathway activity [40,41]. Although direct studies of complement activation in cases of congenital HCMV infection remain limited, findings like these indicate the possibility that mechanisms of the immune system could contribute to the response to gestational viral exposure. In support of this hypothesis, new experimental models that use primary placental cultures and trophoblast organoids were infected with HCMV and have demonstrated that innate immune responses were present [42]. Together, these observations suggest a plausible role complement activation plays in congenital HCMV immunopathology, although further targeted investigation is necessary to define the in vivo extent and subsequent long-term consequences.

HCMV infection has the capacity to activate all three major pathways of the complement cascade. The classical pathway is activated through C1 complex recognition of antigen or antibody-antigen complexes, while the lectin pathway may be triggered by mannose-binding lectin (MBL) that recognizes carbohydrates on the surfaces of several pathogens [14]. The alternative pathway can be activated when the spontaneous activation of a component of the complement system interacts with the surface of infected cells or pathogens. Altogether, these pathways converge and amplify activation, driving subsequent inflammatory signaling in the infected tissues [14].

Excessive activation of the complement during the development of a fetus can initiate harmful inflammatory cascades [43]. This response can cause pathologies such as injury to the endothelium and has the potential to cause disruption to the blood-brain barrier [22]. The immune activation in response to congenital HCMV infection may contribute to the hallmark outcomes, such as FGR and localized injury to tissues [34]. We hypothesize that persistent or dysregulated complement cascade activation and activity could intensify tissue damage during and post-infection with the virus and could play a direct role in the long-term neurodevelopmental sequelae [22].

### 5.2. Known Viral Strategies and Potential Fetal Compartment-Specific Modulators

**Figure 2:**
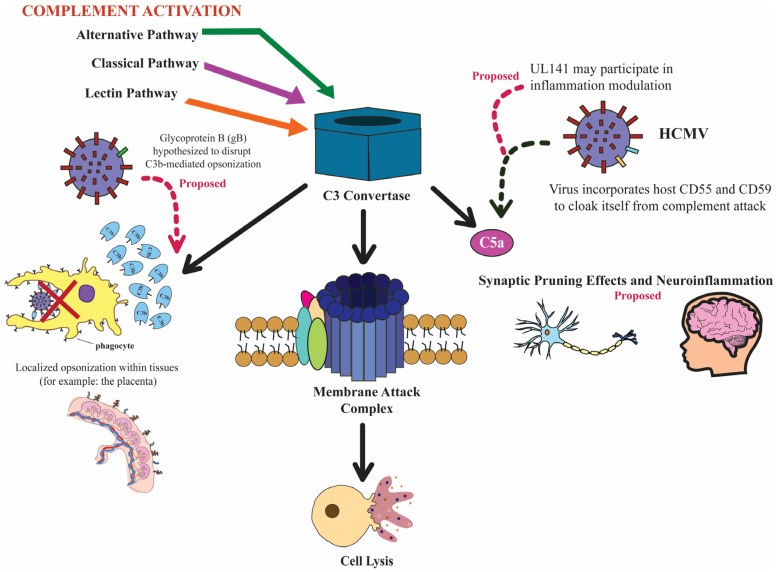
Effects of complement activation on host cells and proposed impacts on the developing fetus, and mechanisms by which HCMV disrupts or evades these responses [14,36,38]. HCMV is suggested to use several strategies, including the incorporation of host regulators (CD55, CD59), possible UL141-mediated modulation of inflammation, and potential interference with opsonization. Downstream impacts on the fetus remain hypothetical and are indicated as such. Solid arrows indicate established complement activation steps; dashed pink arrows represent proposed viral mechanisms; and dashed black arrows represent proposed downstream effects [14,16,38].

Being an obligate intracellular parasite, viruses are found to encode gene products that modulate complement functions and counteract complement-mediated immune responses [14,44]. In particular, being able to establish lifelong infection and co-exist within their hosts, human herpesviruses such as HCMV and herpes simplex virus 1 (HSV-1) have evolved a myriad of strategies to evade complement-mediated immunity. For example, glycoprotein C (gC) of HSV-1 is a well-characterized example of a herpesvirus protein that can bind C3b and interfere with the activity of the complement system [45]. We hypothesize that this evidence could suggest HCMV’s glycoprotein B (gB), encoded by UL55, could have a similar function, even though direct evidence for gB-C3b interaction has not yet been demonstrated. Additionally, HCMV virions have been shown to incorporate host-derived complement regulatory proteins, such as CD55 and CD59, into their envelopes (Figure 2) [46]. This evasion strategy acts as a cloaking mechanism that likely allows the virus to evade immune detection by mimicking host surfaces [14]. Although the study showed that blocking CD55 decreased HCMV titers, there is no direct evidence that this specifically influences MAC formation during HCMV infection. However, a reduction in MAC-mediated lysis is plausible, as it is the terminal component of the complement cascade.

In addition to well-characterized mechanisms, emerging evidence suggests that there are certain HCMV open reading frames (ORFs) that could be responsible for modulating responses of the immune system depending on the context of the tissue [47]. For example, UL141 has been implicated in downregulating both CD155 and TRAIL receptors to help the virus evade natural killer cell-mediated killing. This evidence suggests that UL141 may have additional, currently uncharacterized roles in viral evasion from the complement system within the developing fetal brain [22,47]. Defining tissue-specific effects of these viral factors continues to be an important area of investigation.

The fetal immune environment is highly compartmentalized [37]. To achieve successful infection, HCMV must be able to exploit this system through tailored evasion strategies for entrance into specific tissues [25]. An example of this is in the case of trophoblasts at the maternal-fetal interface. This tissue type expresses high levels of complement regulator proteins [43] and could be modulated differently than in neural tissues, where excessive inflammation has the potential to cause significant, irreversible damage [48]. To gain a better understanding of pathogenesis and viral persistence, it is important to examine the unique strategies employed to regulate the activity of the complement system cascade in response to HCMV infection in these cellular compartments.

## 6. Complement and Neurodevelopmental Outcomes

### 6.1. Disruption of Microglial Pruning and Synaptic Refinement

During development of the fetal brain, as shown in Figure 2, microglia use complement proteins, specifically C1q and C3, to identify weak or excess synapses for elimination through a process known as synaptic pruning [49]. This mechanism is essential for the optimization of efficient neural circuits and helps to ensure appropriate cognitive, sensory, and behavioral functions [50]. We hypothesize that the very complement proteins required for healthy brain development become pathogenic when activated by HCMV, causing damage because their function is so localized to these vulnerable neural tissues. If true, the consequences of unnecessary inflammation in developing tissues could contribute to the observed long-term neurodevelopmental deficits, including impaired sensory processing and cognitive function, associated with congenital HCMV infection.

### 6.2. Injury, Neuroinflammation, and Long-Term Cognitive Risks

White matter abnormalities, ventriculomegaly, and subependymal cysts are hallmark radiologic findings in infant cases of congenital HCMV infection [1,8,51]. These lesions are thought to be reflections of the combination of direct viral cytopathology and immune-mediated injury [22]. Inflammatory complement activation can contribute to vascular injury, demyelination, and blood-brain barrier (BBB) compromise, potentially amplifying tissue damage to fetal developing white matter tracts [52,53]. Evidence of abnormal activation of microglia, via the C3-C3aR axis, has been shown to exacerbate white matter injury in models of chronic brain hypoperfusion [54]. These findings support the hypothesis that complement-driven neuroinflammation could contribute to the neuropathological features that are commonly observed in cases of congenital HCMV infection. The temporal and spatial overlap between complement activation and white matter injury points to a possible mechanistic role for complement in driving neural damage during fetal development.

Persistent activation of complement in the fetal brain could sustain or initiate chronic neuroinflammation, even beyond the period of acute viral infection [55]. Potent anaphylatoxins, like C3a and C5a, recruit and activate infiltrating monocytes and microglia [41]. This creates a pro-inflammatory environment that could disrupt neuronal and glial maturation [56]. In other neuroinvasive viral infections, like HIV, there has been evidence of CD14*^+^*CD16*^+^* monocytes with upregulated expression of CCR2 transmigrating across the BBB [57]. This has been linked to neurocognitive impairment [57]. Based on this parallel, we speculate that in congenital HCMV, excessive complement activation in the developing brain could similarly recruit these damaging monocyte populations, offering a plausible mechanism for the immunopathology observed in neurodevelopmental sequelae.

Additionally, we hypothesize that persistent immune system failure to clear the virus could lead to prolonged inflammatory activation during crucial stages of fetal development and may contribute to long-term cognitive and behavioral sequelae observed in infected children [22]. This elucidates the complement system’s role in recruiting immune cells and could reveal novel therapeutic targets to protect the developing central nervous system during HCMV congenital infection [58].

## 7. Clinical Implications and Therapeutic Potential

### 7.1. Complement as a Diagnostic and Prognostic Biomarker

Complement activation products, including C5a and the soluble terminal complex (sC5b-9), have been identified as potential biomarkers in neonatal and gestational medicine [39,40]. Action by the complement system plays a dynamic and dual role in maternal-fetal health. Activation of the cascade aids in the defense against infectious pathogens and helps to support vascular remodeling [16]. However, overactivation of complement has been linked to several adverse outcomes, including miscarriage, preeclampsia, and preterm birth [16]. The detection of elevated levels of these complement activation products has been reported in inflammation-associated pregnancy complications [16]. These findings could offer diagnostic insight into pathologies observed in cases of congenital HCMV infection. In newborns, the detection of complement activation products could serve as early indicators of neuroinflammatory processes [59]. Such inflammatory activity has the potential to impair essential developmental pathways, with complement markers offering potential as early predictors of adverse outcomes [35]. However, the instability of anaphylatoxins, such as C5a, is a major challenge for reliable use as biomarkers in clinical settings due to their lack of specificity and rapid degradation. Obtaining accurate measurements is highly contingent on the sample collection method and timing, which can make it especially difficult to employ practically.

### 7.2. Complement-Targeted Therapeutics

Therapeutic inhibition of complement, especially at the level of C5 using agents such as eculizumab, has been successfully used to treat conditions such as paroxysmal nocturnal hemoglobinuria (PNH) and atypical hemolytic uremic syndrome (aHUS) [60]. These cases offer real-world evidence of successful and safe gestational use of complement-blocking therapies. With respect to cases of congenital HCMV infection, strategies like these could offer some protection against excessive inflammation that could cause damage to fetal tissues. However, these strategies carry potential risks, including diminished immune control during active viral replication and uncertain long-term consequences for fetal immune development. This underscores the importance of applying such therapies with precision and caution.

One central challenge in complement-targeted therapies during congenital infection is achieving a successful balance between preserving antiviral defense while reducing immunopathology [61]. While excessive complement activity can be harmful to the developing fetus [22,62], given the critical role of the complement system in both the development of the placenta and host defense, overly broad suppression could compromise both maternal and fetal immune competence, potentially diminishing their capacity to contain and control further replication of the virus [43]. Effective treatment will require precise modulation of complement activity rather than broad immunosuppression. Ideally, this approach should be informed by biomarkers capable of more accurately reflecting both disease progression and the status of the immune system. Achieving the right balance is crucial, as the complement system acts both as a necessary immune defense and a potential driver of pathology in congenital HCMV infection [43].

## 8. Challenges and Future Directions

Recent advances in stem cell-derived organoid systems and microfluidic devices offer new tools to bridge the translational gap [63,64,65,66]. Trophoblast organoids enable the study of maternal-fetal interactions and implantation dynamics, while fetal brain and other tissue-specific organoid models allow for observation of tissue-specific immune responses to HCMV infection. Placenta-on-a-chip platforms recapitulate the maternal-fetal interface under dynamic conditions, making them ideal for investigating viral transmission, immune modulation, and complement-mediated injury. These systems create more physiologically relevant models for testing therapeutic approaches [63,64,65,66].

Animal models, especially murine systems, have provided very important insights into gestational development [67,68,69]. However, the murine systems fall short in capturing the specificity and complexity of fetal immune system development, the comprehensive structure of the human placenta, and the cellular tropism of HCMV. Species-specific differences in immune signaling and placental architecture limit their ability to act as a model for complement regulation during pregnancy [67]. These limitations restrict the clinical applicability of current findings and underscore the need for models that can better mirror the complex dynamics of human physiology [67,68].

Guinea pig cytomegalovirus (GPCMV) and rhesus cytomegalovirus (RhCMV) serve as important animal models for studying congenital transmission and immune responses to viral infection [68,69]. GPCMV is the only small-animal model known to allow transplacental transmission of CMV, while RhCMV provides a valuable model for investigating aspects of viral dissemination, immune evasion, and fetal pathogenesis in a primate model [68,69]. Future investigations using GPCMV and RhCMV models will illuminate complement’s involvement in congenital infection and provide supportive insight into its possible roles in HCMV pathogenesis.

We hypothesize that HCMV infection during pregnancy dysregulates the complement system at the maternal-fetal interface, where we speculate that the host’s immune response may become the primary driver of placental injury and adverse outcomes. To dissect these complex interactions, we propose leveraging advanced models that can reveal the specific tropism of both the virus and the complement cascade.

Human pluripotent stem cell-derived organoids offer a three-dimensional platform to study HCMV’s cellular targets and the localized effects of complement activation within a developing tissue. Additionally, microfluidic ‘placenta-on-a-chip’ models can recapitulate the dynamic maternal-fetal interface, allowing for real-time observation of viral dissemination, complement recruitment, and subsequent impacts on placental barrier integrity. These complementary approaches could provide unique, complement-specific insights into HCMV’s high tropism at this crucial anatomical site.

## 9. Conclusions

To advance understanding of the mechanisms of congenital HCMV and its interactions with the human complement system, strong interdisciplinary collaboration is essential. Developmental virologists, immunologists, clinicians, and bioengineers must work to refine existing models and create new systems with a greater ability to replicate the dynamic environment of human physiology during gestation. These coordinated efforts are critical for the next generation of in vitro platforms designed to model transmission, immune modulation, and complement-mediated injury at the maternal-fetal interface. These tools will ultimately guide future therapeutic development aimed at protecting fetuses during their fragile early stages without compromising immune integrity. Future research must, therefore, focus on how HCMV infection tips the delicate balance of the complement system—from a crucial regulator of fetal development and antiviral immunity towards a possible pathological cascade of tissue injury.

## Figures and Tables

**Table 1:** Summary of key complement proteins in pregnancy, including roles in placental development, immune regulation, and pregnancy complications such as preeclampsia. Components include classical pathway proteins (e.g., C1q), regulators (e.g., CD55, CD59), and effectors (e.g., C3a, C5a, MAC). Dysregulation has been linked to inflammation, fetal injury, and adverse outcomes. Data compiled from previous reports [16,36,38].

Protein	Gestational Role	Citation
C1q	Mediator of trophoblast migration and spiral artery remodeling; essential for placental development and immune tolerance.	[16,38]
C3a/C5a	Potent anaphylatoxins that trigger inflammation; implicated in preeclampsia and fetal neuroinflammation; active effector cells.	[36,38]
CD55 (DAF)	Regulator of early C3 activation.	[16,38]
CD59	Inhibitor of MAC formation and terminal pathway; highly expressed on nearly all human cells, including the placenta.	[16,38]
Factor Bb	Elevated levels act as an indicator for increased risk of preterm birth and reported in cases of preeclampsia.	[16,38]
C4b and Soluble C5b-9	Elevated levels are reported in cases of preeclampsia.	[38]
C5	Expressed on the zona pellucida during early embryonic development.	[16]

## Data Availability

Not applicable.
